# Development of multi-frequency impedance scanning electron microscopy

**DOI:** 10.1371/journal.pone.0263098

**Published:** 2022-01-25

**Authors:** Toshihiko Ogura

**Affiliations:** Health and Medical Research Institute, National Institute of Advanced Industrial Science and Technology (AIST), Tsukuba, Ibaraki, Japan; Mohanlal Sukhadia University, INDIA

## Abstract

Nanometre-scale observation of specimens in water is indispensable in many scientific fields like biology, chemistry, material science and nanotechnology. Scanning electron microscopy (SEM) allows high-resolution images of biological samples to be obtained under high vacuum conditions but requires specific sample-preparation protocols. Therefore, there is a need for convenient and minimally invasive methods of observing samples in solution. We have developed a new type of impedance microscopy, namely multi-frequency impedance SEM (IP-SEM), which allows nanoscale imaging of various specimens in water while minimising radiation damage. By varying the frequency of the input voltage signal of the sine wave, the present system can detect dielectric properties of the sample’s composition at nanometre resolution. It also enables examination of unstained biological specimens and material samples in water. Furthermore, it can be used for diverse samples in liquids across a broad range of scientific subjects such as nanoparticles, nanotubes and organic and catalytic materials.

## Introduction

Electron microscopy (EM) has been used to analyse the nano-level structure of organic materials and biological samples [1–4]. However, due to the vacuum inside the EM chamber, liquid specimens require specific sample preparation protocols involving glutaraldehyde fixation, negative staining, cryo-techniques and metal coating or labelling [3, 4]. Therefore, sample holders have been developed to enable observation of samples in liquids under atmospheric pressure [5–8]. In addition, the infrared spectrum microscope [9, 10], Raman spectrum microscope and the electrochemical impedance spectroscope are generally used to analyse sample compositions [11–14]. Recently, the spatial resolution of infrared spectroscopies and Raman spectrum microscopes has been improved and those microscopes can be used simultaneously for observation and sample composition analyses at submicron resolution [9, 11, 12]. In contrast, the electrochemical impedance method measures the impedance of the whole sample and thus cannot provide structural information of the sample [13, 14]. Several groups have reported imaging systems using electrical impedance tomography (EIT) at a few cm resolution [15, 16]. Therefore, development of an electrochemical impedance method that allows observations of nanometre structures will greatly contribute to the analysis of various samples.

Recently, we have developed two original microscope systems, i.e. a scanning electron dielectric microscope (SE-ADM) [17–19] and an impedance scanning electron microscope (IP-SEM) [20] based on a scanning electron microscope (SEM). In SE-ADM, a biological sample or organic material in water is sealed between two silicon nitride (SiN) films in an atmospheric holder [17, 18]. The sample holder is placed in the SEM chamber and an electron beam (EB) irradiates the tungsten (W)-coated SiN film on the top, which causes electrons to be absorbed by the W-coated SiN film, resulting in an electrical potential change. The electrical signal passing through the sample is detected by the metal terminal under the sample holder and imaged [17, 18]. In the IP-SEM system, an input sine wave voltage signal is applied to the metal terminal under the sample holder, and the input signal through the sample is detected by the W-layer on the upper SiN film. The EB scans the W-layer and the image is created by detecting the impedance signals [17, 18]. With our method, it is possible to directly observe biological samples and/or organic materials in liquids without staining. In addition, since the sample is not directly irradiated by the EB, radiation damage is very low [18, 19]. Furthermore, IP-SEM enables us to observe the impedance amplitude images and phase images at a pre-set frequency at a sub-micron resolution [20]. However, our previously developed IP-SEM system allowed us to observe a single frequency image using a single sine wave only. In order to observe the frequency-dependent response of the sample, it was necessary to obtain images of the same area multiple times by changing the input frequency. In addition, multiple EB scans of the same area may cause staining due to the adsorption of carbon and other molecules to the W-coated SiN film.

Here, we have developed an 8-frequency IP-SEM that enables simultaneous observation of 8-frequency impedance images. With this system, input signals of eight different frequencies are mixed and applied to the metal terminal at the bottom of the sample holder. Signals from the W-layer on the upper SiN film detected are separated into eight-frequency signals by four lock-in amplifiers, each of which includes two modules. This system can simultaneously observe eight-frequency amplitude and phase images in a single EB scan. Using this system, we observed polystyrene (PS) spheres and sunscreen samples at a sub-micron resolution. In addition, we analysed the frequency responses of samples by the impedance images.

## Materials and methods

### Eight-frequency IP-SEM system

**Fig 1:**
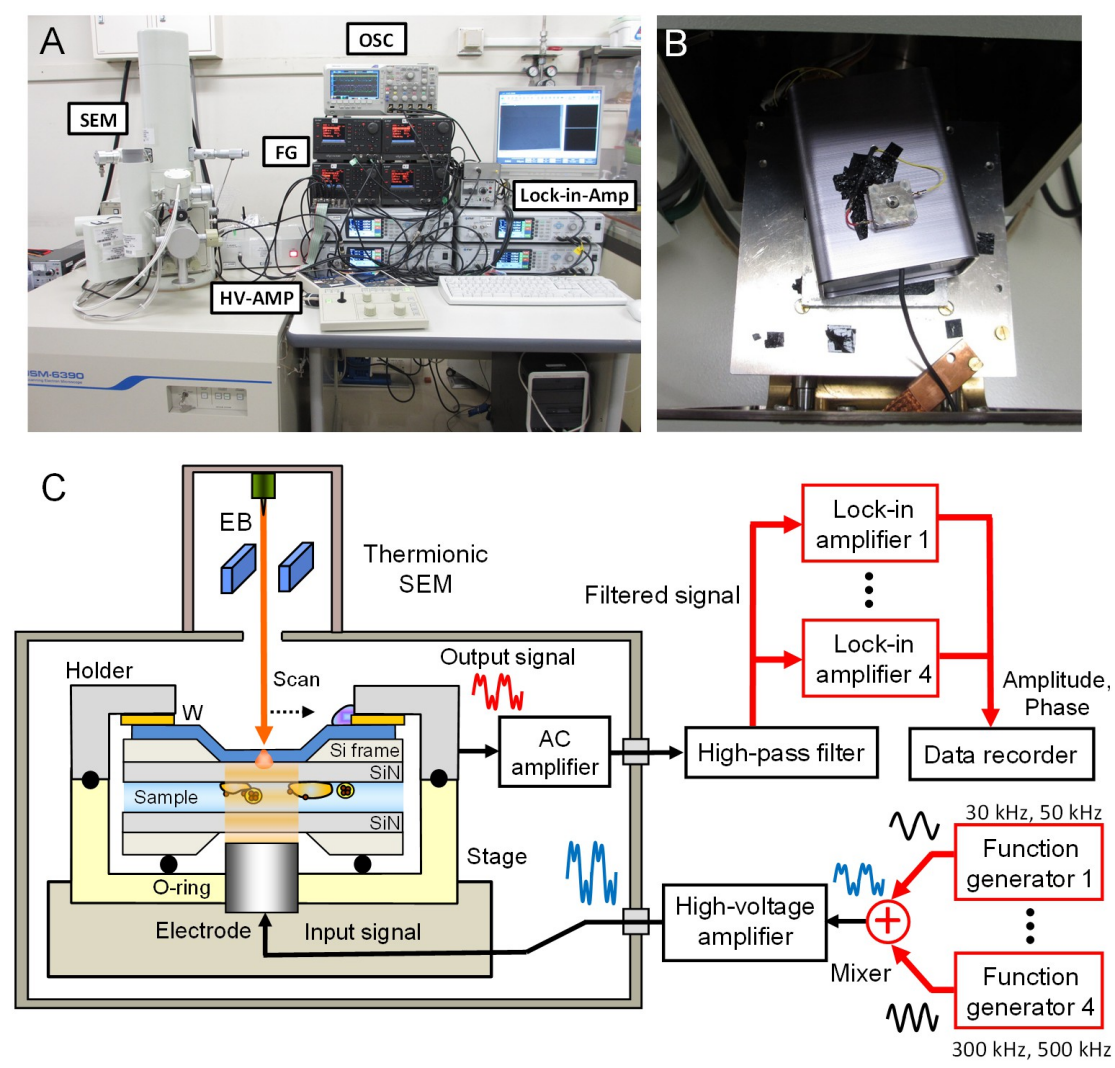
Overview of the 8-frequency IP-SEM system. (A) A photograph of the 8-frequency IP-SEM system, with the SEM on the left side and four function generators (FG), fur lock-in amplifiers (Lock-in-Amp), a high-voltage amplifier (HV-AMP) and an oscilloscope (OSC) for impedance waveform observation on the desk on the right. (B) A photograph of the box containing the preamplifier in the SEM chamber and the sample holder mounted on the box. The red line from the sample holder supplies the output signal from the W-layer of the upper SiN film to the preamplifier. The yellow line connects the bottom aluminium sample holder to the electrical ground. (C) Schematic representation of the 8-frequiency IP-SEM. The 8-frequency signals from the four function generators are mixed and input to the high-voltage amplifier. The mixed signals are introduced to the electrode under the sample holder in the SEM chamber via a connector. The signals from the upper W-layer detected are input to the four lock-in amplifiers after passing through the preamplifier and a high-pass filter. The signals separated into eight frequencies by the four lock-in amplifiers are recorded in two data recorders.

The 8-frequency IP-SEM imaging system based on thermionic-SEM (JSM-6390, JEOL, Japan) is shown in Fig 1A and 1B. The input signal was constructed by sine waves of eight frequencies generated from four function generators, each of which includes two modules (WF1967, NF Corp., Japan), through the mixer (Fig 1C). The mixed eight-frequency signal was amplified 10 or 30 times through a high-voltage amplifier (×10, T-HVA03; ×30, T-HVA02, Turtle Industry Co. Ltd., Japan) and then applied to the metal terminal under the sample holder in the SEM chamber. The output signal was detected from the W-layer on the SiN film after passing through the sample (Fig 1C). This signal was amplified by a preamplifier of the trans-impedance amplifier (200 kΩ gain) located under the sample holder and distributed to four lock-in amplifiers (LI5660, LI5655 and two LI5650, NF Corp., Japan) after passing through a high-pass filter (10 kHz cut off frequency). Each lock-in amplifier has two modules that separate the eight frequencies from the detected signal and output the phase and amplitude of each frequency signal. These output signals and EB scan signals were recorded by two data recorders with 50 kHz sampling frequency (EZ7510, NF Co., Japan). The amplitude and phase waves from the lock-in amplifier during observation were monitored by an oscilloscope (BPO 2024B, Tektronix Inc., USA).

The SEM images (1,280×960 pixels) were captured at ×200 to 10,000 magnification. The scanning time and working distance were 80 s and 7–8 mm, respectively. The EB acceleration voltage was 4 or 7 kV, and the EB aperture was set to be 50–60 at an operating current of 300–500 pA.

### Metal deposition on the upper SiN film

A 50 nm-thick SiN film supported by a Si frame (area, 4.5×4.5 mm^2^ with a 0.4×0.4 mm^2^ window; thickness, 0.381 mm, Silson Ltd., UK) was coated with tungsten using a magnetron sputtering device (Model MSP-30T, Vacuum Device Inc., Japan). Tungsten was sprayed for 10 s under 1.0 Pa Argon with a current of 200 mA, producing a 10 nm-thick coating. The distance between the sputter target and the SiN film was 50 mm.

### Sample preparation

The PS sphere suspension (Micromer 3 μm, Micromod Co., Germany) and trehalose solution were mixed at a ratio of 1:1 to adjust the trehalose weight concentration to 0.1%. For the 0.2% trehalose solution, trehalose (Hayashibara Co. LTD, Japan) was dissolved in ultrapure water. A 5 μL suspension of the PS spheres containing 0.1% trehalose was placed on a SiN film. Then, the suspension liquid was absorbed by a piece of filter paper and dried at 23°C. After drying, spheres attached to trehalose were formed on the SiN film. The Al sample holder with the spheres and trehalose was sealed in the sample holder using four screws and an O-ring [18, 19].

In the case of the sunscreen sample (Sun play super cool, ROHTO Pharmaceutical Co. Ltd., Japan), a 5 μL sample was placed on a SiN film. Then, the Al holder with the sample was placed in a centrifuge holder and centrifuged at 5000 rpm for 1 min (MX300, Tommy Co., Japan) to thinly spread the sample on the SiN film.

### Atomic force microscope system

**Fig 2:**
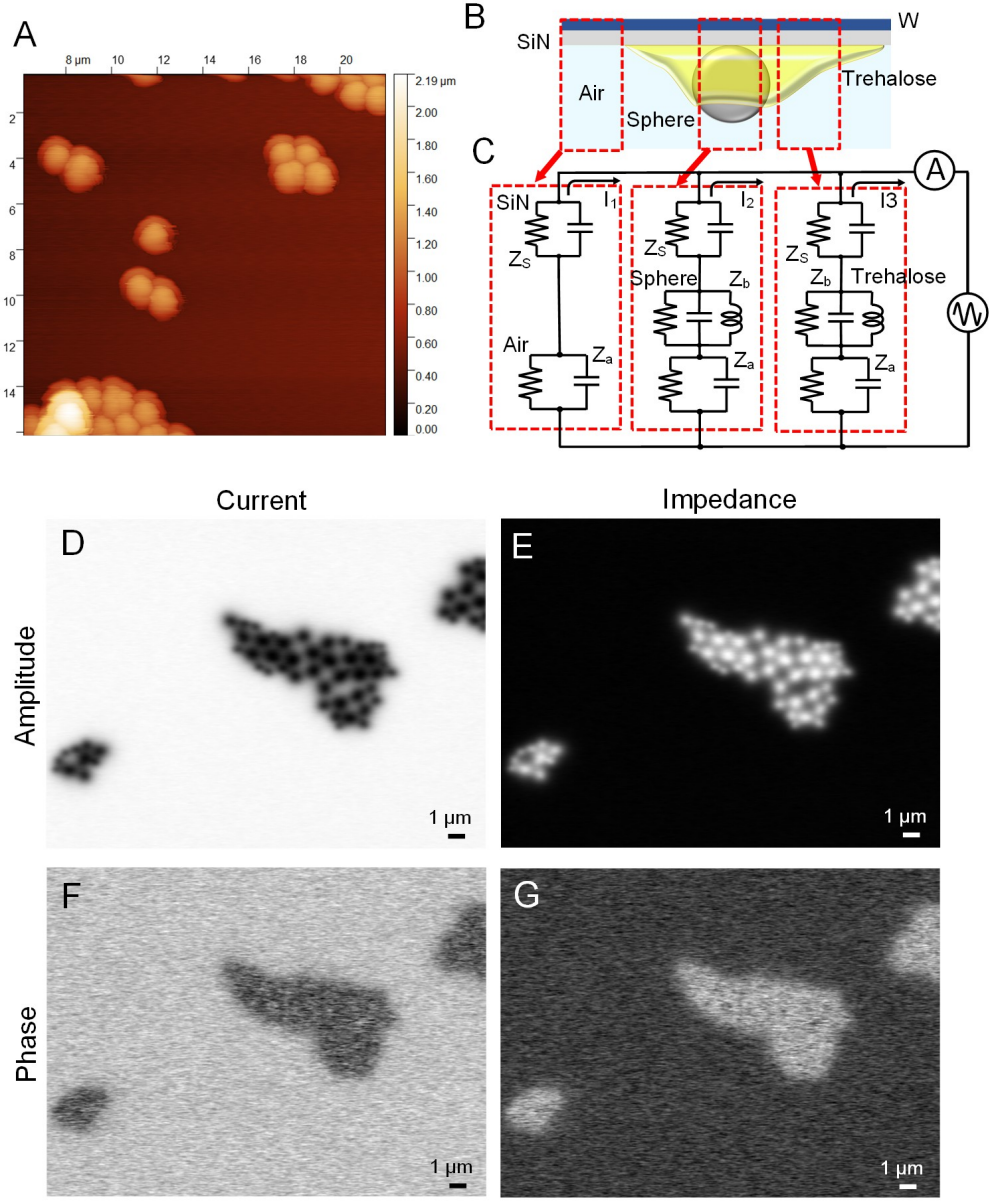
Schematic representation and IP-SEM images of spheres attached to the trehalose layer on SiN film. (A) An AFM image of the PS spheres attached to the trehalose layer on a SiN film. The bottom part of a sphere is embedded in the trehalose layer. (B) Schematic representation of the spheres attached to the trehalose layer. In the IP-SEM observation, the W-coated SiN film is at the top. (C) An electrically equivalent circuit model of the spheres attached to trehalose layer. The upper SiN film consists of resistance R and capacitance C, to which the spheres and trehalose components are connected in series. (D) A current amplitude image of the PS spheres attached to trehalose layer at an input frequency of 30 kHz and at a ×5,000 magnification with 4 kV EB. The current value of the spheres is very small and the spheres are observed as black particles. (E) An impedance amplitude image at an input frequency of 30 kHz of the same field of view as D. The impedance of the spheres is high and the spheres are observed as white particles. (F) A current phase image of the same field of view as D. The trehalose phase is delayed and the spheres are obscure in shape. (G) An impedance phase image of the same field of view as E. The phase of the trehalose layer is advanced. Scale bars, 1 μm in D to G.

An atomic force microscope (AFM) image of a sphere sample shown as Fig 2A was obtained by nGauge AFM system (ICSPI Corp., Canada) with a notebook PC (Panasonic CFZ5, Japan). A PS sphere sample on SiN film was placed on the AFM sample stage and a 15.8×15.8 μm square area was scanned at 512×512 pixels. The scanned data were stored in the notebook PC and background subtraction was performed using Gwyddion 2.56 [21].

### Optical microscopy

The sunscreen sample on SiN film was viewed at ×200 magnification using an optical phase microscope (AXIO Observer A1, Carl Zeiss, Germany). The autofluorescence images of the sunscreen were acquired through a fluorescence filter at excitation/emission wavelengths of 365/420 nm.

### Image processing

The IP-SEM signal data of amplitude and phase stored in the data recorder were transferred to a personal computer (Intel Core i7, 3.8 GHz, Windows 10) and the IP-SEM images were processed by the image processing toolbox in Matlab R2020b (Math Works Inc., USA). The original IP-SEM images were filtered through a two-dimensional Gaussian filter (GF) with a kernel size of 7×7 pixels and a radius of 1.2σ. The background was removed by subtracting the IP-SEM images from the filtered images using a broad GF (kernel size 201×201 pixels, radius 100σ). Finally, amplitude and phase images were converted to 8-bit grey scale format.

### Calculation of the frequency responses of average impedance of the samples

The average impedances of spheres were calculated by the sphere centre of a 5×5 pixel area in the amplitude and phase images at eight-frequencies from 30 to 500 kHz. The number of spheres used in the average calculation was 30, which were manually selected. The trehalose region showed phase advance with the 30 kHz input frequency. The average amplitude and phase values of the trehalose region were calculated from the region greater than 3σ in the phase distribution of the 30 kHz phase image. The average amplitude and phase values of the BG were measured in the area without spheres and trehalose.

## Results

### Eight-frequency IP-SEM system

The eight-frequency IP-SEM based on thermionic SEM that we have newly developed is shown in Fig 1A and 1B. An input signal was created by mixing eight-frequency sine waves of 30 to 500 kHz using the function generators (Fig 1C). The mixed input signal was amplified 10 times by the high-voltage amplifier before being applied to the metal terminal under the sample holder in the SEM chamber (Fig 1C). The input signal passing through a sample was detected by the W-layer on the upper SiN film. The signal detected was amplified by a preamplifier under the stage, passed through an HPF, and then distributed to four lock-in amplifiers each of which has two modules. The output signals of eight-frequencies were simultaneously separated by the lock-in-amplifiers (Fig 1C). The separated 8-frequency signals were recorded by two data recorders and processed by a PC for the impedance images in terms of amplitude and phase.

### Frequency analysis of the sphere impedance using the eight-frequency IP-SEM system

First, we used this system to observe a sample of 3 μm diameter PS spheres (Fig 2). The PS spheres were partially embedded in a thin layer of trehalose attached to the SiN film (Fig 2A). Therefore, the sphere images are classified into three categories: sphere, trehalose, and background of SiN film only (Fig 2B). Fig 2C illustrates the electrically equivalent circuits of these three categories. Trehalose and sphere were connected in series with the SiN film in terms of impedance. When EB was applied to the upper SiN film, the impedance Z_s_ of the SiN film was changed, which caused the current in this area to change (Fig 2C). This current change should represent information about the sample’s impedance. By measuring the change in the alternating current (AC) signal from the terminal under the holder while scanning with the EB, the impedance images of amplitude and phase were calculated using the EB scan signal.

PS spheres attached to trehalose were observed under an 8-frequency IP-SEM. Fig 2D–2G show the current and impedance images of PS spheres attached to trehalose at input frequency of 30 kHz. In the current amplitude image, the spheres were seen to be black, indicating that the current through spheres was very low (Fig 2D). In the impedance amplitude image, the contrast of the PS spheres was reversed because impedance is the inverse of current (Fig 2E). This result indicates that PS spheres are highly insulating, consistent with the actual properties of PS (10^16^ Ω-cm) [22]. In the phase image of the sphere current and impedance, the trehalose region containing the spheres showed a uniform phase and the sphere structures became obscure (Fig 2F and 2G).

**Fig 3:**
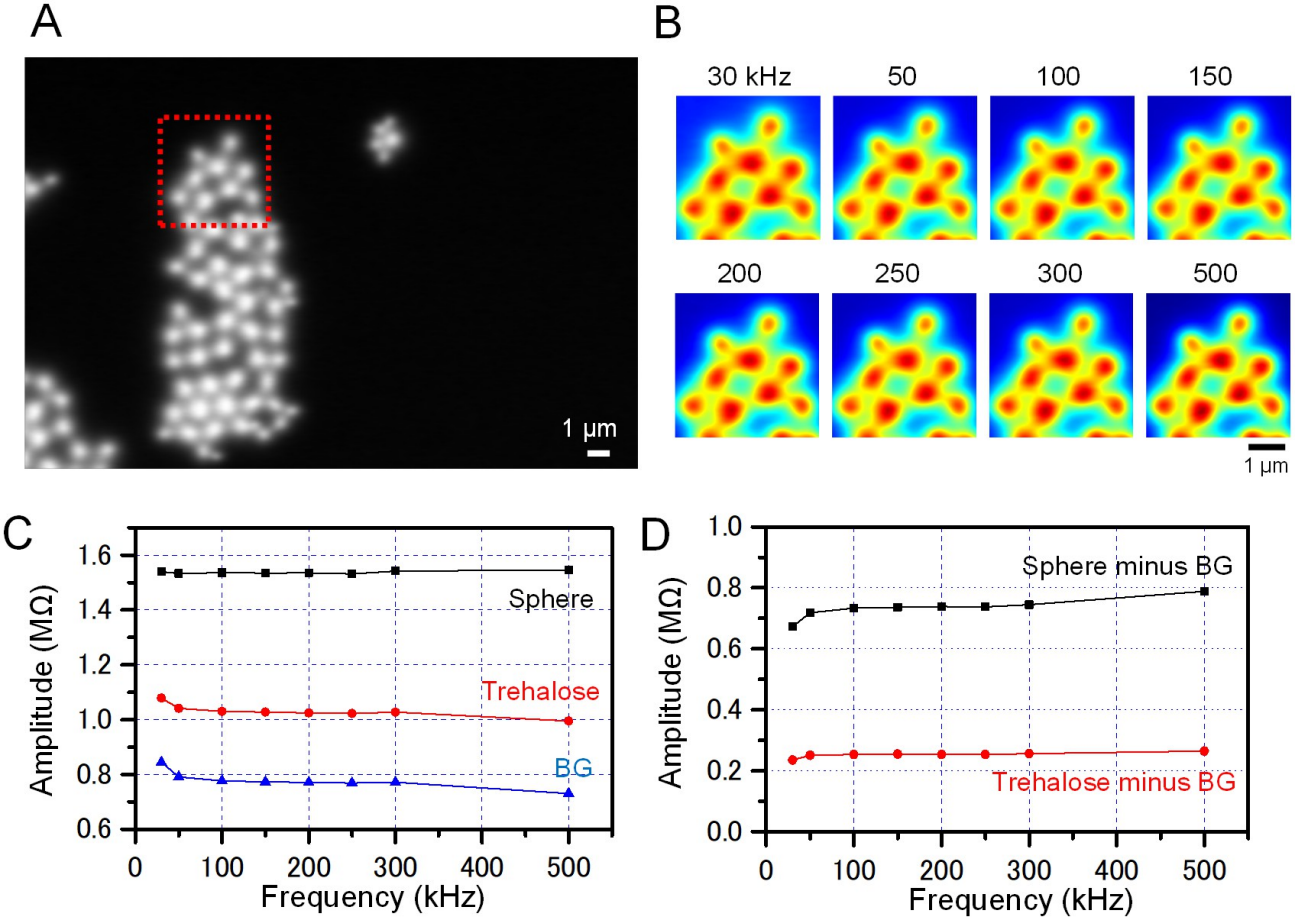
Frequency analysis of the impedance amplitude of the PS spheres attached to trehalose on the SiN film using 8-frequency IP-SEM. (A) An impedance amplitude image at 30 kHz frequency of the spheres and trehalose attached to a SiN film at ×5,000 magnification with 4 kV EB. (B) Enlarged pseudo-colour images of the impedance amplitude at 8 different frequencies of the red square in A. (C) Comparison of the impedance amplitudes of the PS sphere, trehalose and background at various frequencies as indicated on the horizontal axis. (D) Impedance amplitudes of spheres and trehalose after subtraction of those of the background. Impedance of trehalose and spheres increases gradually with increasing frequency. Scale bars, 1 μm in A and B.

**Fig 4:**
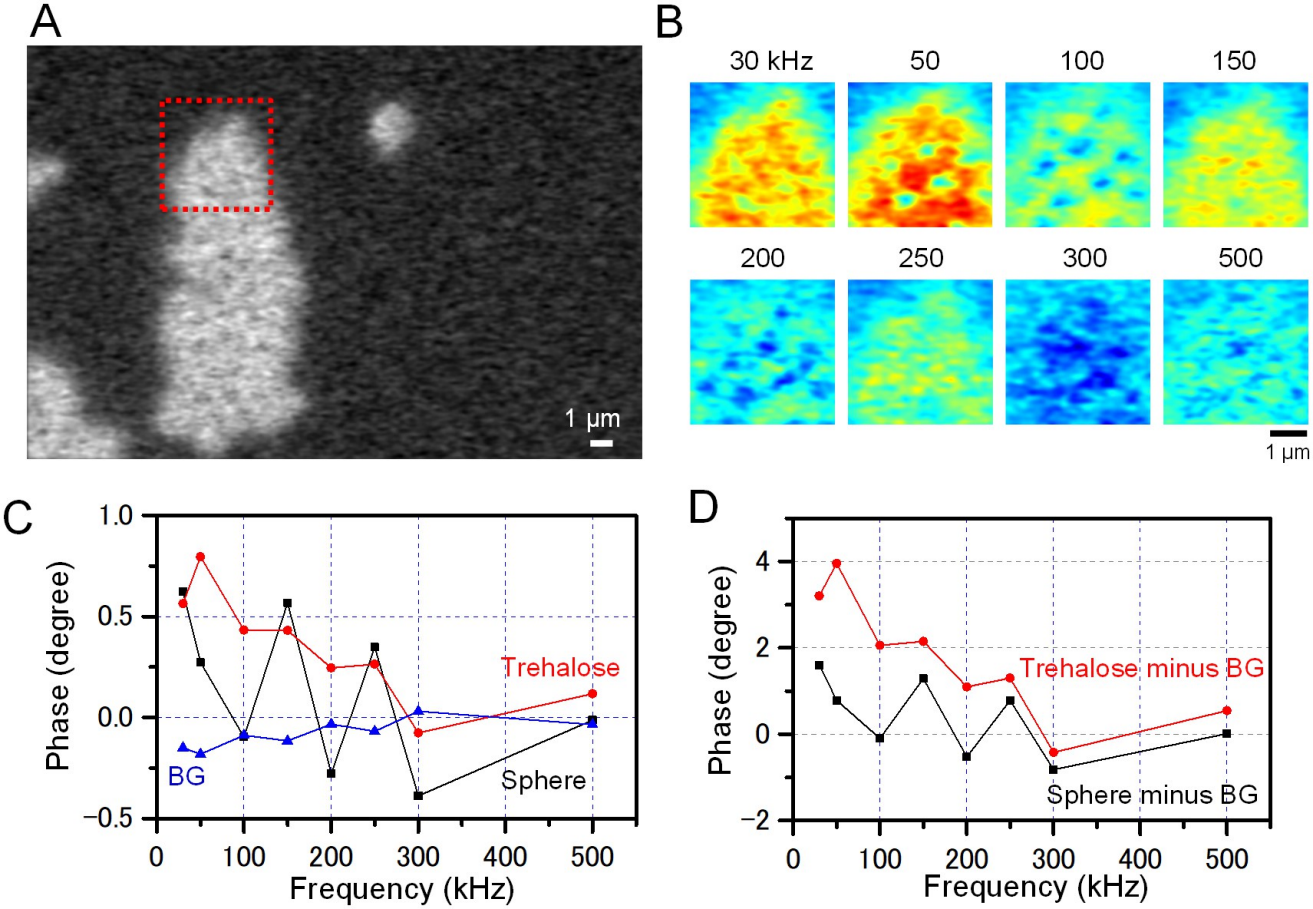
Frequency analysis of the impedance phases of spheres attached to trehalose on a SiN film. (A) An impedance phase image at 30 kHz input frequency of spheres and trehalose attached to a SiN film, the same field of view as Fig 3. (B) Enlarged pseudo-colour images of the impedance phase at 8 different input frequencies of the red square in A. (C) Comparison of the impedance phases of a sphere, trehalose and background at various frequencies indicated on the horizontal axis. The phase of the trehalose advances at low frequencies and gradually delays as the frequency increases. The phase of the sphere shows two peaks at 150 kHz and 250 kHz. (D) Impedance phases of the sphere and trehalose after subtraction of those of the background. Scale bars, 1 μm in A and B.

With another PS sphere aggregate, we analysed the frequency-dependent characteristics of the impedance amplitude and phase (Figs 3 and 4). Fig 3A shows the impedance image of the area of the trehalose-attached sphere aggregate at an input frequency of 30 kHz. In the upper area of the sphere aggregate, the impedance amplitude images at 30 to 500 kHz were very similar irrespective of input frequency (Fig 3B). The frequency dependence of the impedance amplitude at the sphere centre was almost constant, 1.56 MΩ, at input frequency of 30 to 500 kHz (Fig 3C, black line). On the other hand, the impedance amplitude of the trehalose region gradually decreased from 1.1 MΩ to 1.0 MΩ as the frequency increased (Fig 3C, red line). In the background (BG), i.e. in the absence of spheres and trehalose, the value was 0.85 MΩ at 30 kHz, and this value gradually decreased with increasing frequency (Fig 3C, blue line). Since the frequency dependence of BG amplitude affects both spheres and trehalose, the actual impedance characteristics could be obtained by subtracting the BG impedance from those of the spheres and trehalose (Fig 3D). The values obtained by subtracting the BG from the sphere amplitude showed a significant increase from 30 kHz to 100 kHz and then a gradual increase to 500 kHz (Fig 3D, black line). In the region of trehalose after subtraction of BG, the frequency response of impedance amplitude was almost constant (Fig 3D, red line). The impedance amplitude images in Fig 3B indicate little changes with frequency. Because the difference in impedance between the spheres and the background was much larger than the change in impedance with frequency, it is difficult to produce image contrast.

Next, we analysed the phase of the same sphere aggregate (Fig 4). In the phase image at 30 kHz, the contrast of trehalose layer without spheres was observed (Fig 4A and 4B, 30 kHz), suggesting that the phase shift of spheres was equal to that of trehalose (Fig 4C, 30 kHz). In contrast, in the phase images at 50 and 100 kHz, the phase of the sphere region was delayed and the trehalose region was advanced. Therefore, the structure of the spheres in the trehalose layer could be observed (Fig 4B and 4C, 50 and 100 kHz). The sphere structure of this phase image disappeared again at 150 kHz (Fig 4B, 150 kHz), but reappeared at 200 kHz (Fig 4B, 200 kHz). At higher frequencies, it disappeared again (Fig 4B, 250 to 500 kHz).

In the frequency response of phase, the sphere phase sharply delayed between 30 and 100 kHz, and above 150 kHz, two sharp phase changes occurred (Fig 4C and 4D, black line). In the trehalose region, the positive phase was gradually delayed with increasing frequency (Fig 4C and 4D, red line). In BG, the initially negative phase was gradually increased (Fig 4C, blue line). At frequencies of 100 kHz and 200 kHz, where the difference in phase between the spheres and trehalose expanded, the sphere contrast was enhanced (Fig 4B). On the other hand, at 150 kHz and 250 kHz, the phase difference between the two became smaller and the contrast of the spheres disappeared (Fig 4B, 150 kHz and 250 kHz). These phase oscillations of the spheres are thought to be due to impedance resonance [23].

### Frequency analysis of sunscreen impedance using eight-frequency IP-SEM system

**Fig 5:**
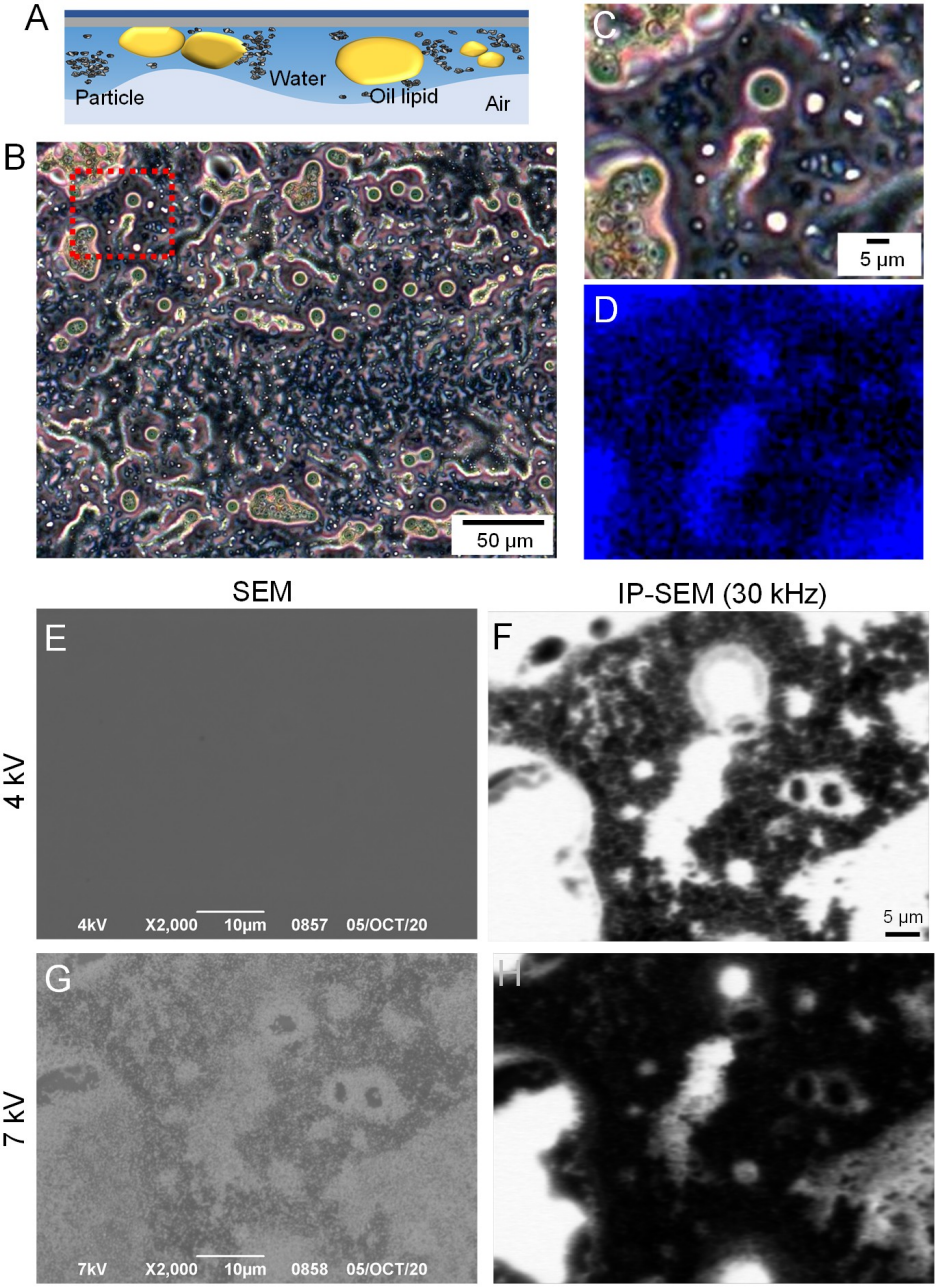
Eight wavelength impedance images of PS spheres and trehalose on a SiN film. (A) Schematic illustration of a sunscreen sample attached to a SiN film. Sunscreens consist of titanium dioxide and zinc oxide particles dispersed in an emulsion of water and oil. (B) An OM image of the sunscreen sample deposited on the SiN film. (C) An enlarged image of the red framed area in B. (D) An autofluorescence image of C using a fluorescence filter at excitation/emission wavelengths of 365/420 nm. (E) A SEM secondary-electron image of the sunscreen sample attached on the SiN film in the same area in C. SEM imaging condition is ×2,000 magnification and at 4 kV EB. The SEM image shows no structures. (F) An image of the impedance amplitude at the frequency of 30 kHz obtained simultaneously as E. The impedance image shows structures similar to those in C. (G) A SEM secondary-electron image of the sunscreen sample at 7 kV EB acceleration. Structures of the sample are observed by the secondary electrons reflected from the sample. (H) An amplitude image at the frequency of 30 kHz at 7 kV EB obtained simultaneously as G. Scale bars, 50 μm in B, 10 μm in E and G, 5 μm in C and F.

The sunscreen sample consists nanoparticles of titanium dioxide and zinc oxide dispersed in an emulsion of water and oil (Fig 5A) [24, 25]. Therefore, it was used in this study for evaluation for our 8-frequency IP-SEM system. A sunscreen sample was placed onto a SiN film, stretched thinly by means of centrifugation, and observed under optical microscopy (OM) and IP-SEM (Fig 5B–5H). A sunscreen OM image showed many oil droplets and small white granules dispersed in the whole field of view (Fig 5B). An enlargement of the red frame in Fig 5B shows a circular oil droplet at the centre and an elongated structure in the bottom (Fig 5C). Fig 5D is an autofluorescence image of Fig 5C. Autofluorescence was observed in the central circular droplet and elongated structures as well as in the peripheral droplets. Since the oil component generally emits autofluorescence [26, 27], it was expected that these structures consisted of the oil component.

In the 8-frequency IP-SEM, our system is capable of capturing impedance images and general SEM images (secondary electron image) simultaneously. When the EB acceleration voltage was 4 kV, only the W-surface on the SiN film was observed in a SEM image (Fig 5E). This indicates that most of the irradiated electrons were reflected and absorbed in the W-coated SiN film but did not reach the sample. In contrast, the impedance amplitude image observed at the same time showed the sunscreen structure clearly under the SiN thin film (Fig 5F). In this image, the high impedance area was almost the same as the oil droplet region of Fig 5D. This agrees well with the fact that oil is a high impedance material. At the acceleration voltage of 7 kV EB, the sample structure was observed by the secondary electrons from the reflected electrons at the sample (Fig 5G). Under this SEM condition, the irradiated electrons penetrated the W-coated SiN film and irradiated the sample slightly. The intensity of the reflected electrons from the sample depended on the sample density, with heavier samples such as metals being observed as a white contrast. In this impedance image, since the impedance was changed by the electrons irradiating the sample, the impedance contrast was reduced compared to that at 4 kV EB (Fig 5H).

**Fig 6:**
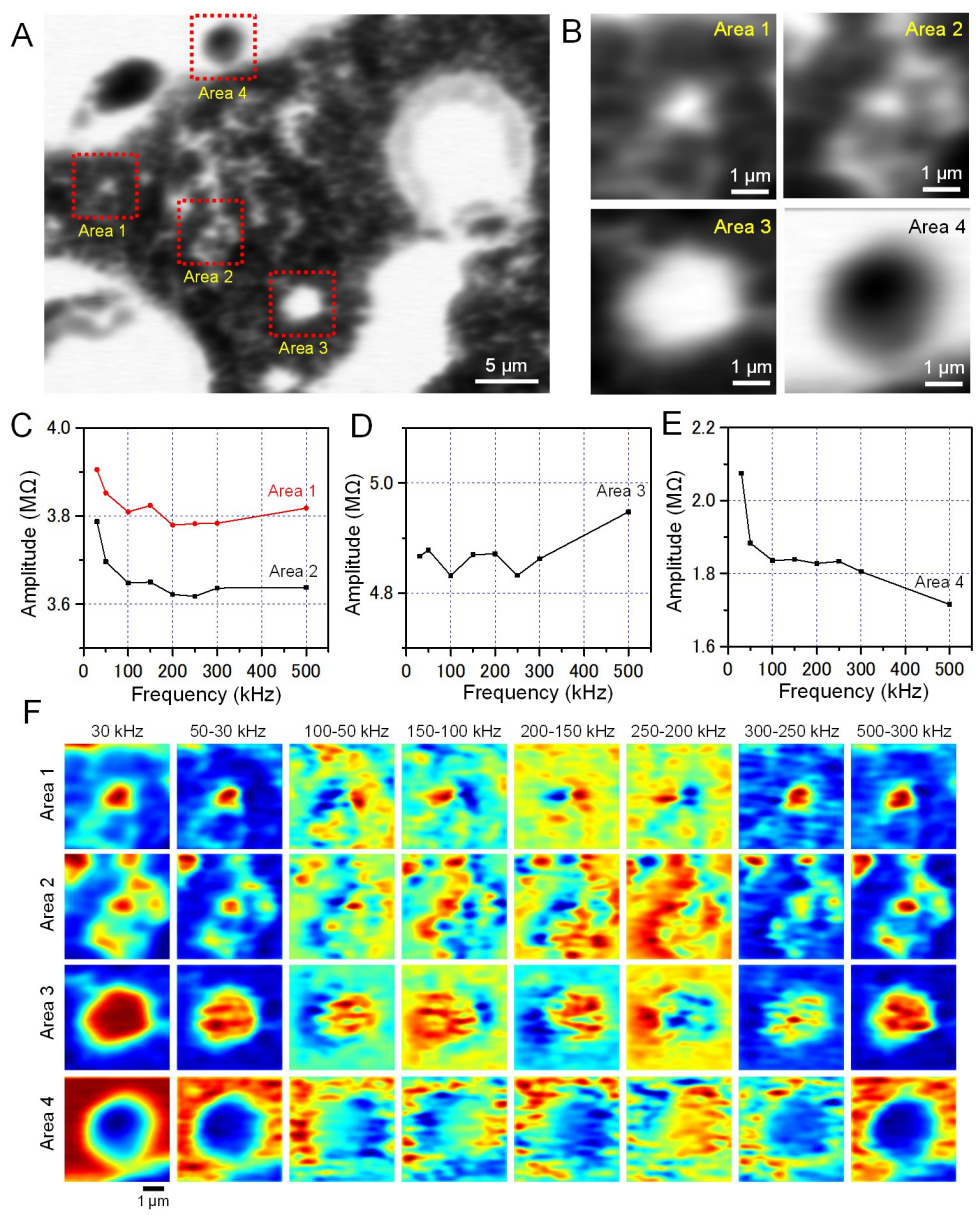
Analysis of the impedance amplitude of the sunscreen sample. (A) An enlarged image of the impedance amplitude at the frequency of 30 kHz of the upper left area in Fig 5F. (B) Enlarged images of the four red squares in A. (C–E) Impedance amplitudes in Areas 1 to 4 at various frequencies indicated on the horizontal axis. (F) Pseudo-colour images of Areas 1 to 4 after subtraction. The leftmost images are the impedance amplitude images of four areas at 30 kHz. The second column images from the left are the impedance amplitudes at 50 kHz after subtraction of the impedance amplitudes at 30 kHz: denoted as 50–30 kHz in Fig 6F. The subsequent columns are the difference images of further frequency pairs. Scale bars, 5 μm in A and 1 μm in B and F.

For detailed analyses of the sunscreen impedance, we calculated the frequency response of the impedance amplitude of the various particles in the image (Fig 6). Fig 6A shows an enlargement of the upper left part of Fig 5F. In this image, many small white particles were dispersed in the central region and the oil area was spread in the peripheral region (Fig 6A and 6B). As for the small particles, Fig 6C shows the frequency response of the impedance amplitude of Area 1 and 2 in Fig 6B. The frequency responses showed that the impedance decreased from 30 to 250 kHz and then gradually increased. For the large diameter droplet of Area 3, the impedance remained at 4.85 MΩ between 30 and 300 kHz and then gradually increased (Fig 6D). In contrast, the amplitude of the low-impedance particle of Area 4 decreased sharply from 30 to 100 kHz, remained constant from 100 to 250 kHz, and then gradually decreased (Fig 6E).

In order to investigate the changes in the images depending on frequency, images showing the differences between the impedance amplitudes of the images obtained at two different frequencies were obtained (Fig 6F). The images on the left side of Fig 6F are the colour maps of the amplitude at 30 kHz. The amplitude images at 50 kHz minus those at 30 kHz (in Fig 6, denoted as 50–30 kHz) showed similar shapes as that obtained at 30 kHz in all four areas (Fig 6F, 30 kHz and 50–30 kHz). The 100–50 kHz images showed different impedance changes on the left and right sides of the particle (Fig 6F, 100–50 kHz). The impedance changes between left and right sides were large, especially for small particles in Area 1 and Area 2. This impedance change was reversed in the 150–100 kHz image pairs, with the left side being positive and the right side being negative (Fig 6F, 150–100 kHz in Area 1 and Area 2). These inversion changes were also seen in the 200–150 kHz and 250–200 kHz images.

**Fig 7:**
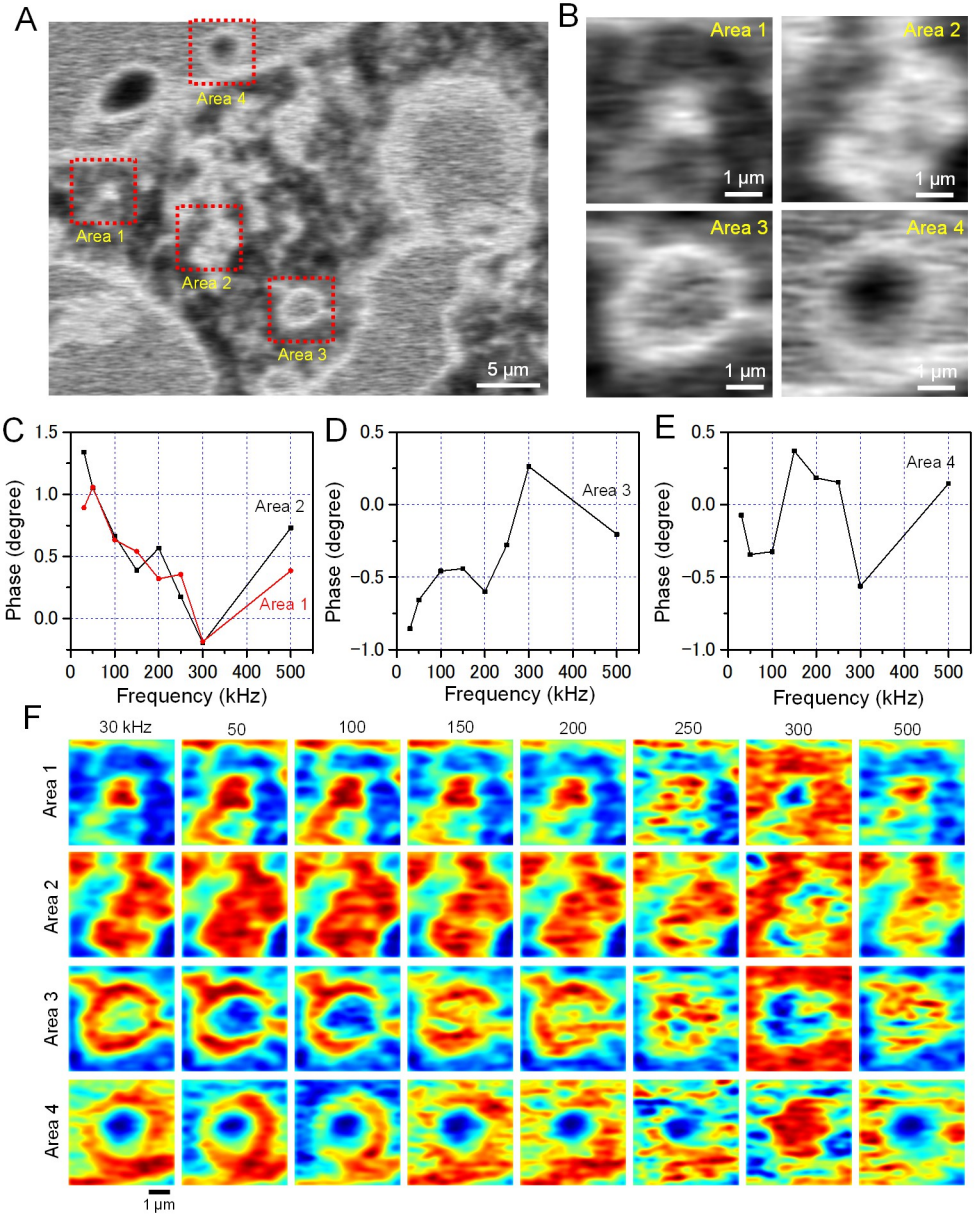
Analysis of the impedance phases of the sunscreen sample. (A) An enlarged phase image at the frequency of 30 kHz in Fig 6A. (B) Enlarged images of the four red squares in A. (C–E) The impedance phase values in Area 1 to 4 at various frequencies indicated on the horizontal axis. (F) Pseudo-colour images the impedance phase values of Area 1 to 4. Each column shows the 8-frequency phase images at 30 to 500 kHz of the four areas. Scale bars, 5 μm in A and 1 μm in B and F.

Finally, we analysed the frequency response of the phase images in Areas 1 to 4 (Fig 7). The phase images of the same region as Fig 7A and the enlarged images of Area 1 to Area 4 are shown in Fig 7B. The phase images of the small granules in Areas 1 and 2 showed almost the same structure as the amplitude images of Fig 6F. In contrast, Areas 3 and 4 were large spherical structures and a positive phase shift occurred at the boundary of the spherical structure, revealing a white ring-like structure (Fig 7B).

The frequency characteristics of the phase in Areas 1 to 4 are shown in Fig 7C–7E. The phase of Areas 1 and 2 showed a linear delay between 30 and 300 kHz and an increase above 300 kHz (Fig 7C). In contrast, the frequency response of Area 3 gradually increased up to 300 kHz and decreased thereafter (Fig 7D). In Area 4, the phase changed up and down within ±0.5 degree (Fig 7E).

The pseudo-colour phase images of Areas 1 to 4 at eight frequencies between 30 and 500 kHz are shown in Fig 7F. In Areas 1 and 2, the phase images were similar to the amplitude images from 30 to 250 kHz. At the input frequency of 300 kHz, the contrast of the image reversed. However, the phase images at 500 kHz returned to the same as the images at 30 kHz (Fig 7F, Areas 1 and 2). Such a contrast inversion at 300 kHz was also observed in Areas 3 and 4 (Fig 7F, Areas 3 and 4). The phase images of the large droplet in Areas 3 and 4 showed ring-like structures at the droplet boundary (Fig 7F, 30 to 100 kHz). Such a phase advance at the boundary suggests that the impedance components of capacitance and reactance at the water-oil interface have drastic changes in the submicron region.

## Discussion

Here, we have developed an 8-frequency IP-SEM system capable of simultaneous observation of 8 frequency images at 30 to 500 kHz (Fig 1). Using this system, we observed PS spheres of 3 μm diameter attached to the trehalose layer on a SiN film (Fig 2A and 2B) at 8 frequencies simultaneously (Fig 3). The PS sphere amplitude gradually increased with increasing input frequency (Fig 3D). The parallel connection of conductance C and resistance R is commonly used as an equivalent circuit for samples. In general, the impedance component consisting of C and R decreases as the frequency increases [14, 28]. Therefore, the increase in the impedance amplitude of the PS spheres suggests a contribution of an inductance component. There are two possible origins for the inductance: the properties of PS as a material or the effect of the interface between PS spheres and trehalose. We plan to do detailed analysis of the inductance component of various samples at a nano-level resolution using detection systems of higher sensitivity.

Next, we analysed the phase characteristics of PS spheres and trehalose (Fig 4). In the background, the phase was slightly advanced, while in the trehalose layer, it was delayed. In contrast, the phase of the spheres showed two sharp peaks at 150 and 250 kHz (Fig 4C and 4D). The spheres were attached to trehalose and it was expected that the impedance components of the spheres and trehalose were connected in series. Therefore, two peaks of the impedance phase suggest that the frequency characteristics of the PS sphere and trehalose are different.

Finally, we observed a sunscreen sample using 8-frequency IP-SEM (Figs 5–7). The sunscreen sample consists of titanium dioxide and zinc oxide particles in a water/oil emulsion [24]. The 8-frequency IP-SEM system enabled us to obtain the impedance amplitude and phase images of water, oil and granules in the emulsion (Figs 5–7).

The frequency images of particles and droplets in the sunscreen sample after subtraction of the background component showed a characteristic left-right contrast change and/or inversion of contrast as the input frequency increased (Fig 6F). At present, it is difficult to determine whether the inversions of impedance amplitude were caused by the characteristics of the sample or by the scanning of the SiN film with the EB. Therefore, we plan to perform further experiments and analyses to determine the origin of the inversions using further developed highly sensitive detection systems. In addition, the present system has a spatial resolution of about 50 nm [20], which makes it difficult to analyse particles smaller than 100 nm in the sunscreen sample. In the future, we plan to introduce a multi-frequency IP-SEM system to a high-resolution field-emission SEM to improve the resolution to be higher than 10 nm.

In conclusion, we have developed an 8-frequency IP-SEM system capable of simultaneously obtaining eight impedance amplitude and phase images from 30 to 500 kHz. Using this system, we observed the impedance of PS spheres and a sunscreen sample. The analysis of the PS sphere showed a frequency response suggesting the existence of inductance components. In the observation of the sunscreen sample, a phase advance phenomenon was observed at the interface between water and oil, which is thought to be caused by a sudden change in impedance at the interface. Our system enables impedance analyses of samples at a sub-micron level. Therefore, this system will contribute to the analysis of biological samples, nano-materials, organic materials and other materials.
